# Binocular mirror–symmetric microsaccadic sampling enables *Drosophila* hyperacute 3D vision

**DOI:** 10.1073/pnas.2109717119

**Published:** 2022-03-17

**Authors:** Joni Kemppainen, Ben Scales, Keivan Razban Haghighi, Jouni Takalo, Neveen Mansour, James McManus, Gabor Leko, Paulus Saari, James Hurcomb, Andra Antohi, Jussi-Petteri Suuronen, Florence Blanchard, Roger C. Hardie, Zhuoyi Song, Mark Hampton, Marina Eckermann, Fabian Westermeier, Jasper Frohn, Hugo Hoekstra, Chi-Hon Lee, Marko Huttula, Rajmund Mokso, Mikko Juusola

**Affiliations:** ^a^Department of Biomedical Science, University of Sheffield, Sheffield S10 2TN, United Kingdom;; ^b^Department of Image Processing and Computer Graphics, University of Szeged, H-6701 Szeged, Hungary;; ^c^Nano and Molecular Systems, University of Oulu, Oulu FIN-90041, Finland;; ^d^European Synchrotron Radiation Facility, 38043 Grenoble, France;; ^e^Xploraytion GmbH, D-10625, Berlin, Germany;; ^f^Department of Physiology Development and Neuroscience, Cambridge University, Cambridge CB2 3EG, United Kingdom;; ^g^Institute of Science and Technology for Brain-Inspired Intelligence, Fudan University, Shanghai 200433, China;; ^h^Key Laboratory of Computational Neuroscience and Brain-Inspired Intelligence, Ministry of Education, Fudan University, Shanghai 200433, China;; ^i^Ministry of Education Frontiers Center for Brain Science, Fudan University, Shanghai 200433, China;; ^j^University of Sheffield Advanced Manufacturing Research Centre, Sheffield S9 1ZA, United Kingdom;; ^k^Institut für Röntgenphysik, Georg August Universität Göttingen, 37077 Göttingen, Germany;; ^l^Deutsches Elektronen-Synchrotron, 22607 Hamburg, Germany;; ^m^Faculty of Electrical Engineering, Mathematics, and Computer Science, University of Twente, UT7522 NB Enschede, The Netherlands;; ^n^Institute of Cellular and Organismic Biology, Academia Sinica, Taipei 11529, Taiwan;; ^o^MAX IV Laboratory, Lund University, SE-221 00 Lund, Sweden;; ^p^National Key Laboratory of Cognitive Neuroscience and Learning, Beijing Normal University, Beijing 100875, China

**Keywords:** compound eyes, stereovision, active sampling, adaptive optics

## Abstract

Neural mechanisms behind stereopsis, which requires simultaneous disparity inputs from two eyes, have remained mysterious. Here we show how ultrafast mirror-symmetric photomechanical contractions in the frontal forward-facing left and right eye photoreceptors give *Drosophila* superresolution three-dimensional (3D) vision. By interlinking multiscale in vivo assays with multiscale simulations, we reveal how these photoreceptor microsaccades—by verging, diverging, and narrowing the eyes’ overlapping receptive fields—channel depth information, as phasic binocular image motion disparity signals in time. We further show how peripherally, outside stereopsis, microsaccadic sampling tracks a flying fly’s optic flow field to better resolve the world in motion. These results change our understanding of how insect compound eyes work and suggest a general dynamic stereo-information sampling strategy for animals, robots, and sensors.

## Significance

To move efficiently, animals must continuously work out their x,y,z positions with respect to real-world objects, and many animals have a pair of eyes to achieve this. How photoreceptors actively sample the eyes’ optical image disparity is not understood because this fundamental information-limiting step has not been investigated in vivo over the eyes’ whole sampling matrix. This integrative multiscale study will advance our current understanding of stereopsis from static image disparity comparison to a morphodynamic active sampling theory. It shows how photomechanical photoreceptor microsaccades enable *Drosophila* superresolution three-dimensional vision and proposes neural computations for accurately predicting these flies’ depth-perception dynamics, limits, and visual behaviors.

Historically, stereovision studies have focused on the disparity between the left and right eye images and how this is processed in the brain (1–7). Less attention has been paid to how the peripheral visual systems actively sample and encode depth information. This trend has been particularly notable with insect vision. Because the insect compound eyes are composed of rigid ommatidial lens systems, it was long thought that their static functional organization provides a pixelated low-resolution image of the world, often with little or no depth information (8, 9).

Remarkably, recent studies have revealed the morphodynamic, active nature of the fruit fly *Drosophila melanogaster*’s early vision in information capture (10, 11). Underneath the ommatidial lenses, light changes make photoreceptors rapidly contract (10, 11) and elongate in and out of their focal plane and sideways in a sophisticated piston motion (11). These microsaccades adjust the photoreceptors’ receptive field sizes and x,y positions dynamically, sharpening light input in time to provide dynamic hyperacute vision beyond the compound eyes’ static optical resolution (11). With phototransduction reactions themselves—PIP_2_ cleavage from the cell membrane (10)—causing the microsaccades, a photoreceptor’s photon sampling itself initiates active vision (11). But it has remained unclear how these microsaccades happen globally, across the left and right eyes, and whether and how they could contribute to visual behaviors and stereovision.

Here, we study how the *Drosophila* photoreceptor microsaccades are organized (adapted) to the world order—its physical regularities—across the two eyes to sample information. We do this first globally, across the left and right eyes of living wild-type and mutant/transgenic fly strains, using ultrafast high-brilliance X-ray imaging (European Synchrotron Radiation Facility [ESRF] and Deutsches Elektronen-Synchrotron [DESY] synchrotrons generating X-ray magnitudes >10^5^ times the conventional X-ray tubes) with electrophysiology. Combined with local high-speed photoreceptor and visual interneuron (large monopolar cell [LMC]) recordings, these results show that photoreceptor microsaccade directions and dynamics are hardwired during development to match the optic flow of a locomoting fly, maximizing visual information capture. Because this active sampling is mirror symmetric between the left and the right eyes, it enables *Drosophila* hyperacute stereopsis. By implementing these experimental results into theoretical multiscale models, we simulate the adaptive *Drosophila* compound eye optics with photoreceptor microsaccades sampling light information across the eyes. Finally, we show how this binocular active sampling theory accurately estimates object depth and predicts various visual behaviors.

## Results

**Fig. 1.**
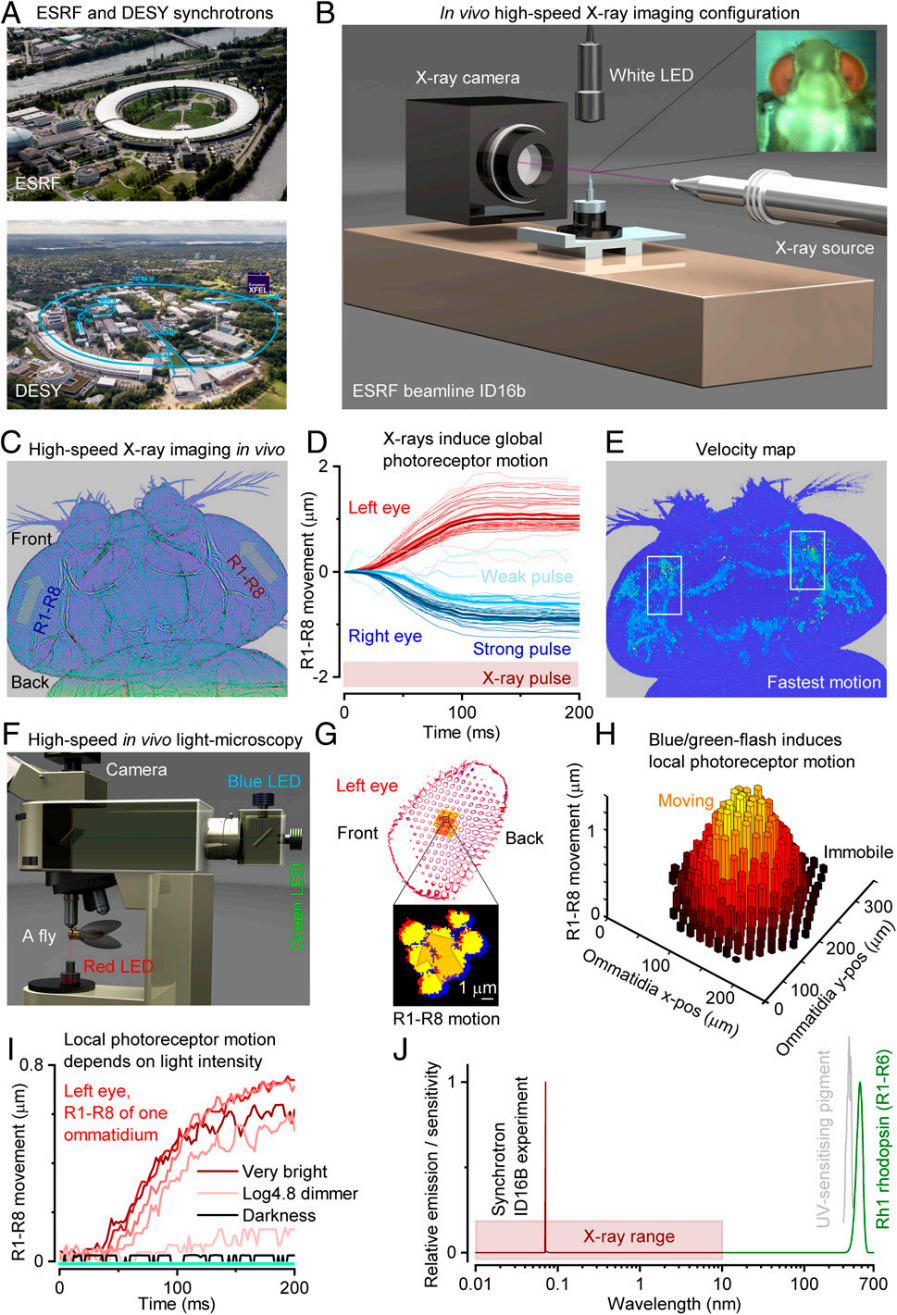
X-ray imaging *Drosophila* in vivo reveals global mirror-symmetric right and left eye photoreceptor contraction dynamics that tie in with local photomechanical photoreceptor responses. (*A*) Experiments were performed using synchrotrons (*SI Appendix*, Figs. S1–S3 and S7–S9). (*B*) ESRF beamline ID16b imaging configuration, using 100-nm resolution. (*C*) X-rays evoked fast synchronized mirror-symmetric photoreceptor (R1–R8) contractions inside the left and right eyes, causing the photoreceptors to sweep in global back-to-front vergence motion (arrows). (*D*) Photoreceptor movement began <10 ms from the X-ray onset, increasing with intensity until saturating. (*E*) The longest frontal forward-facing photoreceptors (15) moved the fastest, ∼15 to 20 µm/s. (*F*) High-speed light microscopy of R1–R8 rhabdomere photomechanics to blue-green flashes under deep-red antidromic illumination (740-nm LED + 720-nm edge filter), with a fly held in a pipette tip. (*G*) A 200-ms blue/green flash, delivered orthodromically (through the microscope optics) into the left fly eye (*Above*), excited local photoreceptors (orange highlight) to twitch photomechanically in a back-to-front direction (arrow). (*H*) Rhabdomeres moved only in the ommatidia facing the incident blue/green flash from above and remained still in the other ommatidia. Thus, R1–R8 motion did not involve intraocular muscles (each eye has a pair) (14), which otherwise would have moved the whole retina (15). (*I*) Local blue/green light–induced photoreceptor movements’ early fast phase depended upon the light intensity and closely resembled those evoked by X-rays (*D*). (*G* and *H*) R1–R8 of one ommatidium contracted together as a unit if any of their R1–R8 alone saw light changes, indicating intraommatidial mechanical photoreceptor coupling; see *SI Appendix*, Figs. S32 and S33. (*J*) The experimental X-ray wavelength peak was ∼6,900 times shorter than R1–R6s’ peak sensitivity (∼480 nm).

To examine the global photoreceptor photomechanics in submicrometer spatial and ≤10-ms temporal resolution inside the compound eyes of intact living *Drosophila*, we performed in vivo X-ray imaging at the ESRF (beamline ID16b) and DESY (beamline P10) synchrotrons (Fig. 1*A* and *SI Appendix*, Fig. S1).

### X-Rays Evoke Mirror-Symmetric Photoreceptor Motion in the Left and Right Eyes.

We first imaged the compound eyes by brief (200 to 300 ms) high-intensity X-ray flashes (Fig. 1*B* and *SI Appendix*, Fig. S2), which would limit radiation damage while simultaneously activating local photoreceptors by a white light-emitting diode (LED) flash, inducing their contraction. Unexpectedly, however, we found that the X-rays alone could rapidly (≤10 ms) activate every photoreceptor to contract in synchrony, causing them to sweep mirror symmetrically inside the left and right eyes in an opposing back-to-front vergence motion (Fig. 1 *C* and *D* and *SI Appendix*, Fig. S3 and Movie S1). This global motion’s size and speed increased broadly with X-ray intensity (Fig. 1*D*) and was large enough to conceal local photoreceptor contractions to the simultaneous LED test flashes. Velocity analyses further revealed that X-rays caused the strongest movements in the left and right eyes’ forward-facing photoreceptor pairs with the longest light-sensitive parts, the rhabdomeres (12), where the photomechanical transduction occurs (10, 11) (Fig. 1*E* and *SI Appendix*, Fig. S3 *E* and *F* and Movie S1).

These movements were not caused by radiation- or heat-induced tissue swelling or damage because immediately, as the X-ray stimulation was shut off in darkness, the photoreceptors stretched back to their original shapes within a second, enabling their contractions to be repeated for many minutes, sometimes ≥30 min. And crucially, the contractions stopped when the fly died and did not appear in freshly killed flies. Moreover, separate light-microscopy experiments through cornea-neutralized ommatidia (Fig. 1*F* and *SI Appendix*, Figs. S31–S33) revealed that 200-ms blue/green flashes (presented within the photoreceptors’ receptive fields) made these cells contract with comparable motion directions (Fig. 1 *G* and *C*), time course and intensity-dependence (Fig. 1 *H* and *D*). These findings suggest that X-rays and visible light elicited the contractions through the same mechanism, requiring phototransduction activation. Interestingly, however, we further discovered that R1–R8 are mechanically coupled in an ommatidium. Activating a single photoreceptor out of R1–R8 within an ommatidium induced them all to contract simultaneously as a unit, without affecting photoreceptors in neighboring ommatidia (Fig. 1 *G*–*I* and *SI Appendix*, Figs. S32 and S33). Thus, the screening pigments around the ommatidia work to insulate the photoreceptors from nonincidental visible light contracting them, but this function fails with X-ray radiation.

We hypothesized that sufficiently high X-ray photon densities could either activate phototransduction directly through rhodopsin photoisomerization (13, 14) or release visible photons through Compton scattering from the heavier atoms inside the eye (15), for example, from phosphorus in the membrane phospholipids or radiation phosphene (16). Such low-energy photons would then photoisomerize rhodopsin molecules or be absorbed by ommatidial screening pigments, preventing light from leaving the eye. The probability of an X-ray photon (λ_x_ ≈ 0.07 nm) activating a single rhodopsin molecule (Rh1, λ_max_ ≈ 330 [UV-sensitizing pigment] and 480 nm [blue-green]) should be infinitesimal (Fig. 1*J*). Yet, each photoreceptor has millions of rhodopsin molecules and faces ∼10^6-8^ X-ray photons in the synchrotron beam at each second. In these extreme conditions, rhodopsin photoisomerizations—and the subsequent fast PIP_2_ cleavage from the photoreceptor membrane, as the plausible mechanism of photoreceptor contractions (10)—may become unavoidable.

### X-Ray–Activated Phototransduction Uncovers Global R1–R8 Microsaccade Dynamics.

**Fig. 2.**
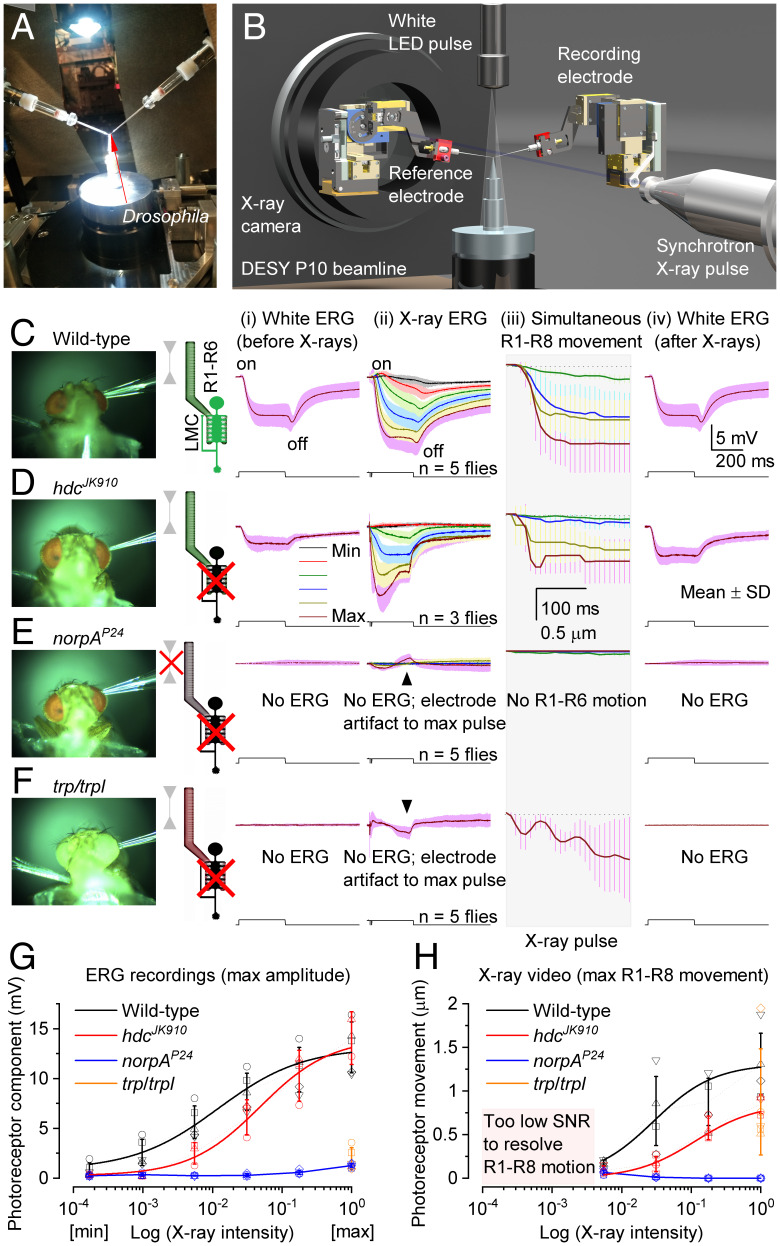
X-rays activate phototransduction. (*A*) Remote-controlled stimulation and recording system for head-fixed *Drosophila*, including two piezo micromanipulators, an ERG amplifier and a white LED, fitted in the DESY P10 beamline. (*B*) Microelectrodes recorded the fly eyes' combined response, ERG, to white-light and X-ray pulses. (*C*) Wild-type ERGs to a white light (*i*) and X-rays (*ii*) show on and off transients, indicating normal histaminergic synaptic transfer. Hyperpolarizing photoreceptor ERG component and (*iii*) R1–R8 photomechanical contraction increased with X-ray intensity. (*iv*) White-light ERG control recorded 20 s after the X-rays. (*D*) *hdc^JK910^* (*i*) white and (*ii*) X-ray ERGs lacked on and off transients, indicating missing synaptic transfer. (*ii*) ERG photoreceptor component and (*iii*) R1–R8 photomechanics increased with X-ray intensity. (*iv*) *hdc^JK910^* white ERG control recorded 20 s later. (*E*) Blind *norpA^P24^* do not generate ERG responses or photomechanical photoreceptor contractions to white-light or X-ray pulses. (*F*) Blind *trp;trpl* do not generate ERG responses while their photoreceptors contract photomechanically to white or X-ray pulses but in a less coordinated way. (*C*–*F*) In the R1–R6/LMC cartoons, green indicates the normal function, gray R1–R6 no contraction, and the black LMC no synaptic output. (*G*) Wild-type and *hdc^JK910^* ERG photoreceptor components increased sigmoidally with X-ray intensity (following a logistic dose–response function), while those of *norpA^P24^* and *trp;trpl* did not respond. (*H*) Wild-type and *hdc^JK910^* photomechanical responses grew sigmoidally with X-ray intensity (here approximated with Michaelis–Menten kinetics), while those of *norpA^P24^* did not respond. The maximal X-ray–induced photoreceptor contraction in *trp;trpl* (orange) was comparable to the wild type and *hdc^JK910^*. (*G* and *H*) The normalized maximum intensity corresponds to 2.2 × 10^6^ photons/s/μm^2^.

We tested this hypothesis in vivo by recording wild-type and blind mutant (*hdc^JK910^*, *norpA^P24^*, and *trp;trpl*) flies’ global electrical responses, so-called electroretinograms (ERGs), to 250-ms white-light and X-ray flashes (Fig. 2 and *SI Appendix*, Figs. S4–S6 and Movie S2) at DESY beamline P10 (Fig. 2*A*). These experiments, measuring retina-wide simultaneous photoreceptor activations, were performed by a remote-controlled LED stimulation/ERG recording system (Fig. 2*B*), synchronized with 100 fps high-speed X-ray imaging, after carefully positioning a recording microelectrode on the right eye and a reference electrode in the thorax and letting the flies dark adapt for 1 to 2 min.

Wild-type white-light control ERGs (Fig. 2 *C*, *i*) showed a typical hyperpolarizing photoreceptor component between on and off transients from the postsynaptic interneurons (17), LMCs. Remarkably, the test ERGs to progressively intensified X-ray flashes (Fig. 2 *C*, *ii*) recorded 20 s after showed comparable dynamics, suggesting that X-rays activated phototransduction, causing an electrical photoreceptor signal and its synaptic transmission. The photoreceptor component increased with the X-ray intensity, consistent with normal elementary response (quantum bump) integration (11). For the two brightest X-ray flashes, this component was larger than the white-flash one, presumably because the X-rays activated every photoreceptor in the eye (global activation). In contrast, the white LED activated mostly the photoreceptors directly facing it (local activation). Importantly, high-speed imaging (Fig. 2 *C*, *iii*) showed that the X-ray–evoked photoreceptor contractions closely followed their ERG dynamics (Movie S2), supporting the direct phototransduction-activation hypothesis. The robust control ERGs (Fig. 2 *C*, *iv*) recorded after the X-rays implied that the eyes worked normally with little (or no) radiation damage.

*hdc^JK910^* mutant ERGs (Fig. 2*D*) gave further evidence that visible light (Fig. 2 *D*, *i*) and X-rays (Fig. 2 *D*, *ii*) activated phototransduction analogously. Both types of stimuli evoked photoreceptor components but no on and off transients, consistent with *hdc^JK910^*-photoreceptors’ inability to synthesize neurotransmitter histamine and transmit visual information to LMCs and the brain (18). While the *hdc^JK910^* phototransduction approximates wild type (11, 18), histamine deficiency has been shown to cause an excitatory synaptic feedback overload from the lamina interneurons to R1–R6s, making *hdc^JK910^* photoreceptors more depolarized with faster responses and reduced light sensitivity with respect to the wild type (18) (compare Fig. 2 *D*, *i* and *iv* to Fig. 2 *C*, *i* and *iv*). Accordingly, and in further support of our hypothesis, we found both the *hdc^JK910^* X-ray ERG dynamics (Fig. 2 *D*, *ii*) and photomechanical contractions (Fig. 2 *D*, *iii*) faster and less sensitive than in the wild type (Fig. 2 *C*, *ii* and *iii*) over a broad intensity range (Fig. 2 *G* and *H*).

Conversely, *norpA^P24^* mutants, in which faulty phospholipase-C molecules halt phototransduction PIP_2_ activation (10), showed (Fig. 2*E*) neither clear electrical responses to visible light (Fig. 2 *E*, *i*) or X-rays (Fig. 2 *E*, *ii*), producing effectively flat no-change ERGs (bar the small electrode charging artifacts), nor photomechanical reactions (Fig. 2 *E*, *iii*) over the test intensity range (Fig. 2 *G* and *H*). Although similar “zero-response” controls were recorded from freshly killed flies (by freezing) (*SI Appendix*, Fig. S5*C*), concurrent X-ray imaging revealed that *norpA^P24^* mutants were alive and active during the stimulation, seen by their antennal movements and intrinsic muscle activity. Thus, these results validated that the wild-type (Fig. 2*C*) and *hdc^JK910^* X-ray responses (Fig. 2*D*) were not caused by tissue shrinkage, damage, or movement artifacts but resulted from phototransduction activation.

Finally, we used *trp;trpl* mutants (Fig. 2*F*), which can respond photomechanically to light flashes by cleaving PIP_2_’s bulky headgroup (InsP_3_) from the microvillar membrane (10) but not electrically because they lack the light-gated ion channels, which are required to open for generating electrical responses and synaptic signaling. Thus, these mutants provided a decisive test of whether the X-ray–induced photoreceptor movements (Figs. 1 and 2 *A*–*E*) were photomechanical. However, owing to their minutes-long light recovery time (11), we used only one bright X-ray intensity. We found that *trp;trpl* mutants neither responded electrically to white-light (Fig. 2 *F*, *i* and *iv*) nor X-ray flashes (Fig. 2 *F*, *ii*), but their photoreceptors contracted strongly both to X-rays (Fig. 2 *F*, *iii*) and visible light (10, 11), meaning these movements were photomechanical and induced by phototransduction PIP_2_ cleavage. And while their dynamics showed characteristic oscillations after contracting ∼40 to 50 ms (11), these were unrelated to missing eye-muscle activation (each eye has a pair) (19). This is because, in the head-fixed wild-type flies, the local photoreceptor activation (Fig. 1*F*) did not trigger intraocular muscle contractions (Fig. 1*G* and *SI Appendix*, Figs. S32 and S33), and yet their local and global photomechanics ensued alike (compare Fig. 2*C* to Fig. 1 *D* and *H*). Therefore, the *trp;trpl* oscillations more likely reflected suboptimal Ca^2+^ dynamics (missing Ca^2+^ influx), mechanical damping/anchoring, or both.

These results (Figs. 1 and 2) showed that a *Drosophila* photoreceptor responds to both X-rays and visible light but with different probabilities and that the synchrotron-based X-ray imaging activates all photoreceptors inside the left and right eyes at once, revealing their photomechanical mirror-symmetric motion dynamics (Movies S1 and S2), hidden from the outside view. Interestingly, these global R1–R8 microsaccade dynamics suggest that when experiencing contrast variations in natural scenes, the two eyes’ frontal forward-facing photoreceptor pairs, which are ∼400 µm apart but should have overlapping receptive fields (RFs), would scan over the same small visual area in opposing but synchronized vergence motion. We, therefore, next asked whether the frontal photoreceptors sample the world in this way?

### Left and Right Eye Photoreceptor Receptive Fields Move Mirror Symmetrically.

**Fig. 3.**
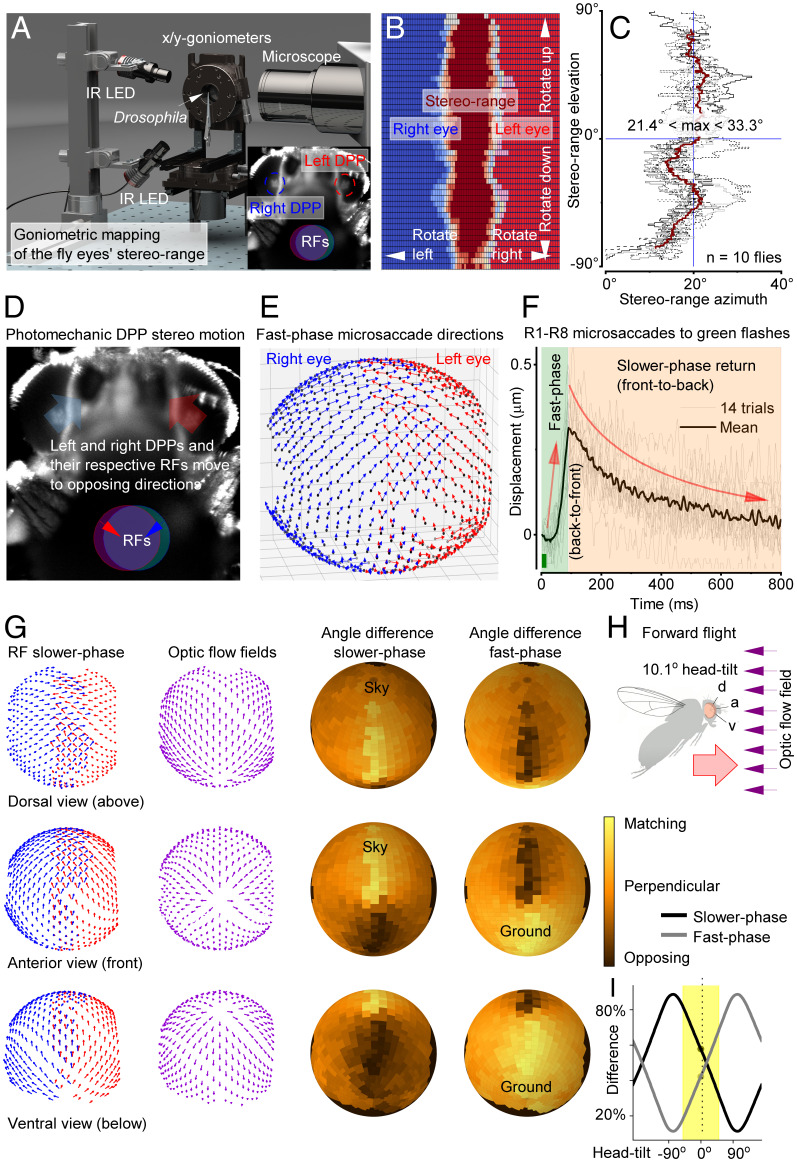
Left and right eye photoreceptor RFs overlap frontally and move mirror symmetrically, tracing forward translation-induced optic flow. (*A*) A goniometric high-speed imaging system for mapping photoreceptors’ RFs. *Inset*: Infra-red (IR) back-lit R1–R7/8 photoreceptor rhabdomeres, forming the left and right eye DPPs (23) (circled), ∼10× magnified by the ommatidial lenses. Each eye’s DPP shows rhabdomeres from neighboring ommatidia that collect light from overlapping RFs (superposition). (*B*) Rotating the fly head through its central x,y axes revealed its DPPs’ stereoscopic visual field (vine color); see Movie S3. (*C*) Because the frontal photoreceptors’ RFs overlap binocularly (∼23.5° azimuth, ∼180° elevation), these mirror-symmetric pairs could enable depth perception. (*D*) Ommatidial lenses invert the eyes’ fast up medially recoiling microsaccades (DPP fast phase; big arrows), evoked by a 10-ms light flash within their overlapping RFs, to sweep their respective RFs down laterally (small arrows). (*E*) Microsaccade fast-phase directions mapped across the left (red) and right (blue) eyes; slower-phase return in the opposite direction (compare Movie S4; mean of 5 ♂ flies). (*F*) Brightening (10-ms light flash) contracts R1–R8 front to back (fast phase), and darkening returns them back to front (slower phase); their RFs move in the opposite directions. The mean (black) and 14 consecutive R1–R7/8 contractions (light gray), recorded through cornea-neutralized optics (compare Fig. 1*G*); see *SI Appendix*, Figs. S23 and S24 for fully light-adapted dynamics (Movie S5). (*G*) The corresponding slower-phase RF vector map (*Left*) compared to the forward flying fly’s optic flow field (*Center*), as experienced with the fly head upright. Their difference (error) is shown for the slower and fast phases. The fast phase matches the ground flow, the slower phase the sky flow. (*H*) By adjusting microsaccadic sampling to optic flow through head tilt, a fly can actively keep the passing world longer within its photoreceptors’ RFs, which theoretically should improve acuity to resolve the world in motion (*SI Appendix*, Figs. S56–S61 and Movie S6). d, dorsal; a, anterior; v, ventral viewpoints. (*I*) Upright (0°) head, and normal tilting around this position (yellow), keep RFs’ fast and slower phases in a balanced push–pull sampling state. Optimizing vision for specific behaviors, like object tracking, requires further self-adjustments in locomotion speed and head and body movements (Movie S7 and *SI Appendix*, Figs. S25–S27).

To answer this question, we built a head-centered goniometric two-axis rotation stage with an integrated microscope/high-speed camera system for targeted rhabdomere light stimulation and motion capture (Fig. 3*A* and *SI Appendix*, Fig. S10). This device allowed us to measure a head-fixed *Drosophila*’s photoreceptor rhabdomeres’ x,y positions in situ (Fig. 3 *B*–*D* and *SI Appendix*, Figs. S11–S14), as visualized by their virtual images, so-called deeppseudopupils (DPPs) (20), to antidromic infrared illumination (≥820 nm, propagating through its head/eyes), which the flies cannot see (11, 21). Moreover, to capture their photomechanical contractions (Fig. 3 *E* and *F*), the rhabdomeres could be stimulated orthodromically, through the ommatidial lens system, with light flashes presented at their RFs.

We first identified those frontal photoreceptors in the left and right eyes, which had overlapping RFs (Fig. 3*B* and *SI Appendix*, Figs. S13 and S14) by systematically mapping their x,y positions (Fig. 3*C*) with head-centric fine rotations (0.35° step) (Movie S3). These measurements revealed the eyes’ stereoscopic layout, where owing to the eyes’ optical superposition design (20, 22), a single point in space frontally is seen at least by 16 photoreceptors; the R1–R8 superpositioned in the left eye and the R1–R8 superpositioned in the right eye (Fig. 3 *B* and *C*). We further mapped how R1–R8 rhabdomeres, as revealed by the DPP images, were systematically rotated during ontogenic development for each eye location while retaining optical superposition with the changing eye curvature. This scanning revealed the left and right eyes’ highly ordered mirror-symmetric R1–R8 angular orientation maps, with equatorial mirror symmetricity (23) between the eyes’ upper and lower halves (*SI Appendix*, Figs. S11 and S12).

Next, we analyzed the rhabdomeres’ photomechanical movement directions to UV or green-light flashes (Fig. 3*D* and *SI Appendix*, Figs. S15–S30), as delivered at their RFs (Movie S4). The resulting deep-pseudopupil microsaccades were then translated into a three-dimensional (3D) vector map (Fig. 3*E*), covering the frontal stereo section and more peripheral parts of the eyes. Expectedly, the left (red) and right (blue) eye microsaccades were mirror symmetric. But crucially, by comparing these movement maps to the deep pseudopupil angular orientation maps for each eye location (*SI Appendix*, Fig. S12), we found that the local microsaccades occurred along their R1–R2–R3 photoreceptors’ rotation axis, implying that their sideways movement directions were hardwired during development. Moreover, because DPPs are virtual images (23), which are magnified but not inverted by the ommatidial lens system (Movie S4 and *SI Appendix*, Figs. S15–S19), the rhabdomeres inside the eyes recoiled accordingly (Fig. 3*F*); first bouncing along their location-specific back-to-front directions (fast phase) before returning front-to-back (slower phase), consistent with the X-ray–imaged photoreceptor movements (Fig. 1*C*). Therefore, during the light stimulation, the corresponding photoreceptor RFs—inverted by the ommatidial lenses (11)—scan the visual world with the same two phases but in the opposite directions (Fig. 3*D*).

Remarkably, the global 3D vector map of photoreceptors’ photomechanical RF movement directions (Fig. 3*G*, red and blue arrows and *SI Appendix*, Fig. S25) sweep along a forward flying/walking fly’s optic flow field (purple arrows), which radiates from a focus at its apparent destination, curving around its left and right eyes. Their difference maps (yellow matching; black opposing) are shown for a characteristic upright head position (Fig. 3*H*) for both the fast and slower phase. Generally, the fast phase is in the flow field direction and the slower phase in the opposite direction (Movie S6). But keeping the head upright sets the RFs’ fast and slower phases in a balanced midstate (Fig. 3*I*), where the fast phase matches the “ground flow” and the slower phase the “sky flow” (Fig. 3*G*). However, locomotion among real-world structures (24) would further burstify sampling (11) in a push–pull manner (Fig. 3*F*). Across the eyes, photoreceptors inside each ommatidium would uniquely and orderly ripple between the phases, as incident light increments drive their RFs fast backward and light decrement slower forward, with some moving patterns thus staying longer than others within an RF, which should improve their neural resolvability/detection in time (11). Thus, the fast ventral components may improve, resolving complex visual clutter, and the slow dorsal components, the landscape and clouds in the skyline. Rotation (yaw) further enhances binocular contrasts (11), with one eye’s fast and slower phases moving with and against their rotation, respectively, while simultaneously the other eye’s phases do the reverse (Movie S7 and *SI Appendix*, Figs. S25–S27).

Control experiments confirmed the fast microsaccades purely photomechanical (*SI Appendix*, Figs. S15, S20–S23, and S28–S36) and similar in both sexes (*SI Appendix*, Fig. S16), reaffirming their phototransduction origin, and validated the X-ray data (Fig. 2). Accordingly, the synaptically decoupled *hdc^JK910^* photoreceptor microsaccades (18) traced the wild-type trajectories (*SI Appendix*, Fig. S22) set by their matching rhabdomere orientations (*SI Appendix*, Figs. S11 and S12). Moreover, the microsaccades adapted to light contrast changes much like voltage responses (*SI Appendix*, Figs. S23 and S24), with different spectral photoreceptor classes’ microsaccades scaling with their ERGs (*SI Appendix*, Figs. S28–S30 and Tables S2–S5). These results show that microsaccadic sampling along the local small-field motion axes initiates optic flow processing (25) and suggest that such sampling and locomotion behaviors have jointly evolved to the physical world order to maximize visual information.

### L2 Interneurons’ Hyperacute Motion Sensitivity Tracks Microsaccade Directions.

To test directly whether the optic-flow–tuned microsaccadic sampling improved acuity of moving stimuli directionally, as suggested experimentally (Fig. 3 *E*–*G*) and predicted theoretically (11), we recorded neural responses of specific LMCs, L2 interneurons (Fig. 4 and *SI Appendix*, Figs. S38–S46), to moving bars and panoramic black-and-white gratings, in which resolution, velocity, and direction were changed systematically.

**Fig. 4.**
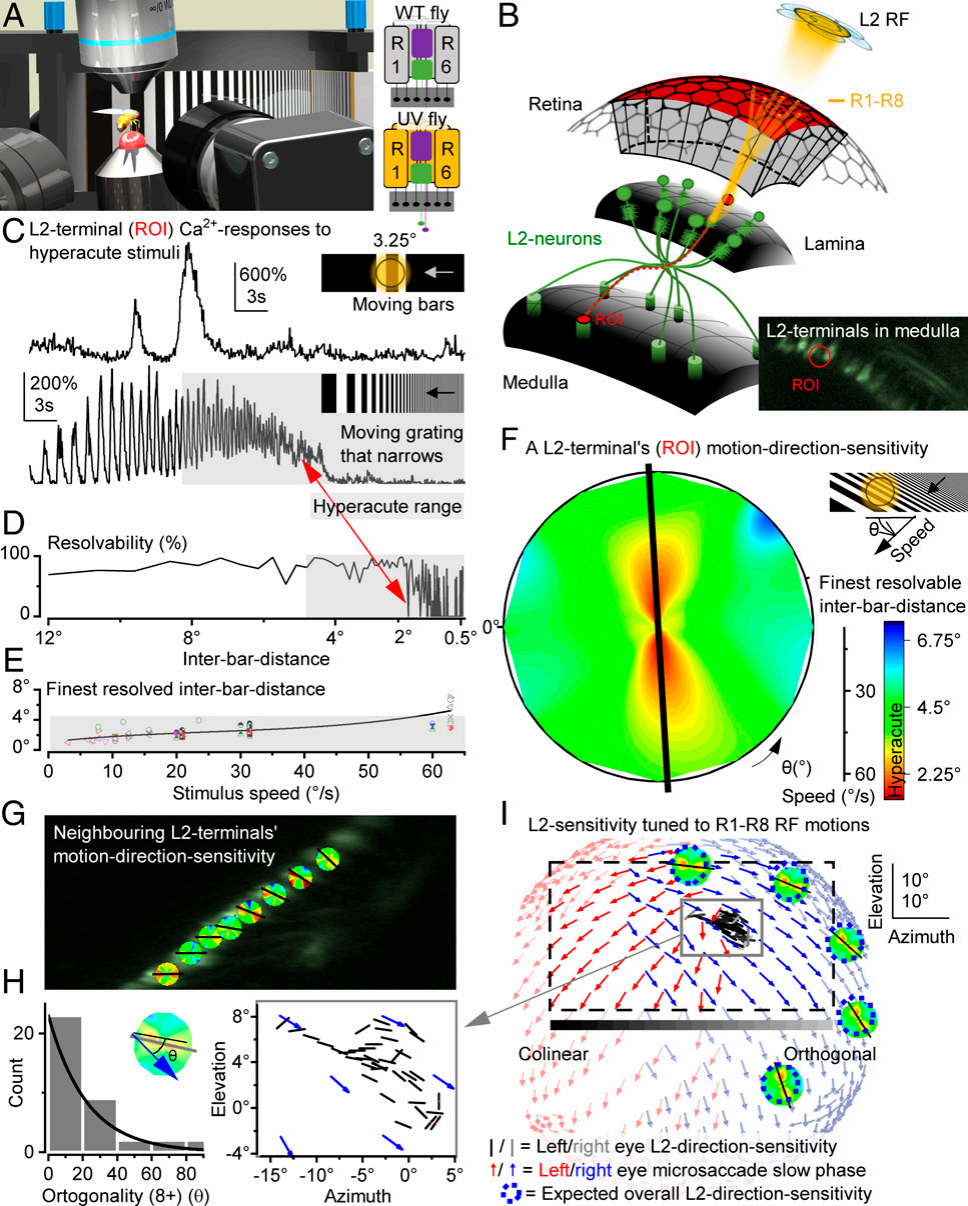
Hyperacute L2-terminal sensitivity follows microsaccade directions. (*A*) A UV fly saw ultrafine (∼0.5°-pixel resolution) UV stimuli on a 150° × 50° screen 38 mm away, while its L2 neurons’ GCaMP6f-fluorescence changes (Ca^2+^ responses) were recorded by high-speed two-photon imaging. In UV flies (25), UV-sensitive Rh3-opsin is expressed in R1–R6s, containing nonfunctional Rh1-opsin (*ninaE^8^*). (*B*) Each L2 RF samples information from six optically superimposed R1–R6 RFs. L2 retinotopy through axonal crossing: Distal lamina L2s project terminals to the frontal medulla. *Inset*: Single L2-terminal Ca^2+^-fluorescence responses to UV stimulation were analyzed as regions of interest (ROI) (red). (*C*) L2-terminal responses resolve in time hyperacute moving bars (here, showing a larger second-bar response) and black-and-white gratings (interbar distance <4.5°, gray), crossing their RFs, over a broad range of orientations and velocities. (*D*) L2 resolvability for a dynamically narrowing grating, moving 20.9°/s. Red arrow indicates the finest resolvable angle (interbar distance, as a rounded-up conservative estimate). (*E*) Interbar distance resolvability depends on stimulus velocity. L2s’ GCaMP6f readout resolved hyperacute patterns moving 60°/s. Note, the finest L2 resolvability, ∼1.09°, approaches the visual display’s two-pixel limit (∼0.5° pixels) and that L2 voltage can encode even faster/finer inputs (34, 35). (*F*) An L2 terminal’s motion-direction sensitivity map is broadly hyperacute, here primarily along the vertical axis (the black line shows its fitted orientation tuning). The map shows the finest resolvable interbar distances to a dynamically narrowing moving grating stimulus (*C*–*E*), covering 360° directions at different speeds. (*G*) Neighboring L2 terminals show a gradual shift in their dominant motion-direction sensitivity (black arrows; see *SI Appendix*, Section IV for analytical details). (*H*) *Drosophila*'s combined L2-terminal motion-direction sensitivity map for the tested left eye region shows retinotopic organization (*Left*, *n* = 4 flies), mainly colinear to the corresponding left eye microsaccade directions (*Right*, compare Fig. 3*E*). (*I*) Eye-location–specific L2-terminal direction sensitivities map R1–R8 microsaccade directions. Thus, L2 terminals collectively generate a high-resolution neural representation of the moving world, enhancing visual information transfer during forward locomotion. The dotted rectangle specifies the visual area covered by the display screen.

These recordings were primarily done in so-called ultraviolet (UV) flies (21), using a bespoke two-photon Ca^2+^-imaging system (Fig. 4 *A* and *B*), while presenting UV stimuli in an ultrafine spatiotemporal resolution to a fly walking on a trackball (*SI Appendix*, Figs. S38 and S39). R1–R6 photoreceptors of UV flies express only Rh3 (UV rhodopsin), and therefore see UV but not green (21), while their L2 neurons express the green-fluorescent Ca^2+^-reporter GCaMP6f. Critically, UV flies show normal photomechanical microsaccades (*SI Appendix*, Fig. S37) and, as their L2 green-fluorescence Ca^2+^ responses cannot activate the UV-sensitive R1–R6s through orthodromic green-light transmission (21), they enable naturalistic low-noise conditions for recording high-precision neural signals (Fig. 4 *C* and *D*). Even so, the wild-type eye L2-GCaMP6f controls’ Ca^2+^ responses showed consistently similar general dynamics, and thus both results were pooled (Fig. 4*E*).

We found that L2 neurons robustly respond to hyperacute 1 to 4° moving gratings with location-specific velocity and motion direction sensitivities (Fig. 4 *C*–*E* and *SI Appendix*, Figs. S40 and S41 and Movie S8). Thus, by encoding spatial information in time, akin to photoreceptors (4), L2s can transmit finer image details than the compound eye’s optical limit, 4.5° interommatidial angle (12) (Fig. 4*F* and *SI Appendix*, Fig. S41*C*), improving vision. Moreover, the angular maximum of L2 response acuity shifted systematically between neighboring medulla terminals (Fig. 4 *G*–*I* and *SI Appendix*, Figs. S42 and S43), showing that directional motion information from microsaccadic photoreceptor sampling was retained at the medulla input layer. Crucially, the L2 terminals’ motion-sensitivity map was essentially colinear to the photoreceptor microsaccade direction map (Fig. 4 *H* and *I* and *SI Appendix*, Fig. S44), indicating angular conservation of synaptic information from R1–R6 to L2 (off channel) LMCs, consistent with preserving the downstream optic flow processing (25). Future experiments need to test whether this is also true for L1 (on channel) and L3 (26–28) LMCs, as asymmetric microanatomical adaptations (29–35) may further influence local motion computations.

These results demonstrate that L2s collectively convey a high-resolution neural representation of the moving world, maximizing visual information flow (Fig. 3*E*).

### Binocular Microsaccades Provide Hyperacute Depth Information.

**Fig. 5.**
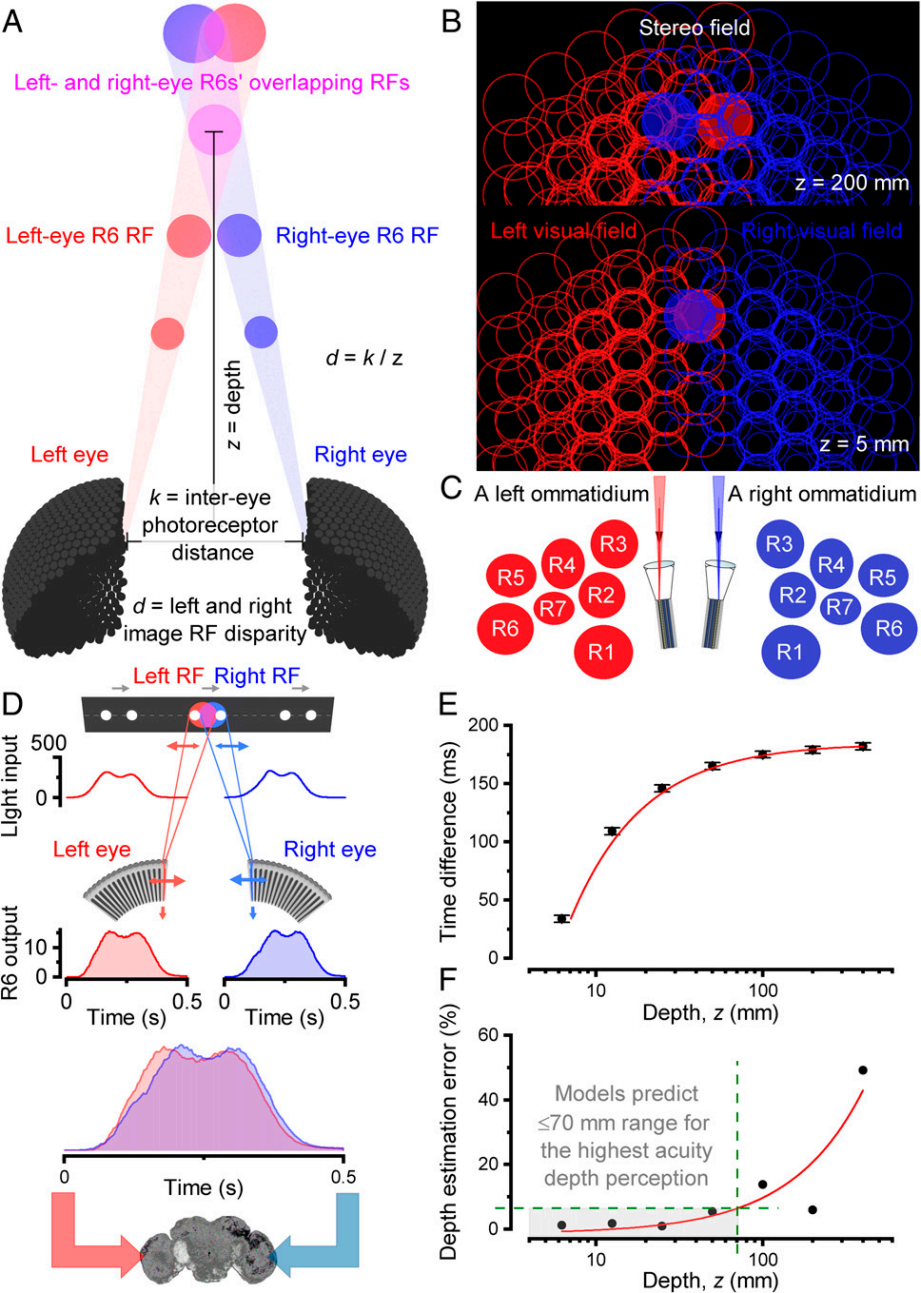
Forward-facing binocular photoreceptors' biophysically realistic multiscale modeling predicts phasic motion disparity for hyperacute stereopsis. (*A*) With the corresponding left- and right-eye photoreceptors being a fixed distance, *k*, apart, their RF disparities inform about the object depth, *z*. (*B*) R1–R8’s beam-traced (40) RFs (half-width circular cuts of broadly bell-shaped functions; right eye, blue; left eye, red) tile the fly's visual fields over completely; shown at virtual planes 200- and 5-mm depths from the eyes. (*C*) R1–R7/8 rhabdomeres of each paired left and right ommatidia lay mirror symmetrically (compare Movies S3 and S9). Because rhabdomeres are of different sizes (11) and distances away (22) from the ommatidium center, so too are their projected RFs (*B*). Therefore, in the neural superposition pooling, the resultant R1–R7/8 RFs do not overlay perfectly into one 4.5° pixel (classic view), but instead tile over completely each small area in the eyes’ visual fields. (*D*) Phasic voltage response differences of binocularly paired photoreceptors enhance object resolvability in time and carry information about the object depth, *z*, to the fly brain (*SI Appendix*, Figs. S55–S61). Two dots, 3.5° apart moving left to right at 50°/s, cross binocular RFs of the corresponding left and right R6s 25 mm away. The resulting mirror-symmetric microsaccades make the RFs move along (right R6) and against (left R6) the passing dots, shaping their light inputs and voltage outputs (Movie S10). (*E*) The proposed binocular mirror-symmetric microsaccadic sampling model (*SI Appendix*, Fig. S59) translates the depth of a moving object into the distance in neural time. The closer the object to the fly’s eyes, the shorter the time difference between the responses. Error bars indicate stochastic jitter. (*F*) The model predicts that *Drosophila* cannot estimate the depth of more distant objects accurately. The error is >10% when an object is >100 mm from the fly eyes, comparable to their distance discrimination estimate (2 to 200 mm) (41).

By comparing two neural images generated by the left and right eye forward-facing photoreceptors, a fly may extract depth information from the corresponding left and right RF pairs’ (“pixels”) x,y-coordinate differences. This disparity, *d*, is inversely related to the scene depth, *z* (Fig. 5*A* and Movie S9). By applying ray tracing from the ommatidial lenses to the world (*SI Appendix*, Figs. S47–S61), with parameters taken from their rhabdomeres’ Fourier transform beam-propagation simulations (36) and 100-nm resolution X-ray imaging (Fig. 1), we first estimated how the corresponding RFs at varying distances from the eyes, and their combined visual field, would look like if the photoreceptors were immobile (Fig. 5*B*).

#### Static case.

The mirror-symmetric sampling array of the paired left and right eye ommatidia (Fig. 5*C*), in which each R1–R7/8 rhabdomere is a different size (11) and distance (22) from the ommatidium center (*SI Appendix*, Figs. S50 and S54), leads to overlapping RF tiling over the frontal stereo field (Fig. 5*B* and *SI Appendix*, Tables S1 and S6). Each eye’s spatial sampling matrix is further densified by the neural superposition signal pooling between seven neighboring ommatidia, in which R1–R7/8s’ RFs of different sizes stack up unevenly (*SI Appendix*, Figs. S57 and S58). This massively overcomplete sampling array greatly differs from the classically considered organization (8, 9), where each ommatidium was considered a sampling point, or a pixel, with a *Drosophila* seeing the world through ∼880 such pixels, giving poor spatial resolution with marginal stereopsis. In contrast, our simulations, using the real R1–R7/8 rhabdomere spacing and sizes (Figs. 1–3), imply that its left and right eyes’ RF overlap disparity could accentuate frontal resolvability and stereo vision.

But how would the frontal RFs and their neural responses change during photomechanical microsaccades? Furthermore, given that these are left–right mirror symmetric (Figs. 1–4), could their phase differences to rotation be exploited for dynamic triangulation (Fig. 5*A*) to extract depth information in time about the real-world distances and relative positions?

#### Dynamic case.

To simulate how the *Drosophila* left (red) and right (blue) eyes probably see left-to-right moving objects, we set their frontal photoreceptors in their respective model matrixes to contract mirror symmetrically to light changes (Fig. 5*D*, two left-to-right moving dots) along with the measured dynamics (Fig. 3 and *SI Appendix*, Figs. S23 and S55). These caused their respective RFs (red and blue disks) to narrow and slide in and out of each other in opposing directions, phasically shaping their neural responses (*SI Appendix*, Figs. S56–S61 and Movie S10), as calculated by biophysically realistic *Drosophila* photoreceptor models (*SI Appendix*, Figs. S53–S55) (11, 37, 38). The responses for the left RFs, which moved against the object motion, rose and fell earlier than the responses for the right RFs, which moved along the objects and so had more time to resolve their light changes. Such phase differences in time broadly correspond to the case where similar but not identical images are sequentially presented to each eye, allowing a fly to perceive 3D space.

Importantly, R1–R8s’ size differing, moving, narrowing, and partially overlapping RFs, with stochastic R7/R8 rhodopsin choices (39) and R1–R6 microstructural/synaptic variations (11, 29) make the retinal sampling matrix stochastically heterogeneous (*SI Appendix*, Figs. S57 and S61). This should eliminate spatiotemporal aliasing in early neural images (11). Therefore, theoretically, this dynamic sampling can reliably feed the fly brain with 3D hyperacute information flow. In the centers interlinking the binocular inputs (40), such as the lobula complex (41–43) (*SI Appendix*, Fig. S62), the distance of an object crossing the corresponding left and right eye photoreceptor RFs could then be represented as distance in time (Fig. 5*E* and *SI Appendix*, Fig. S59). To velocity normalize these distance estimates, their corresponding response waveforms could be correlated with those of their near neighbors (*SI Appendix*, Fig. S59 and Movie S10). These results imply that neural motion and depth computations innately mix, as they share the same input elements, being consistent with the neurons of the motion detection channels serving vision and behaviors more broadly (40, 44) than just specific reductionist ideals.

### Visual Behavior Confirms Frontal Hyperacute Stereopsis.

**Fig. 6.**
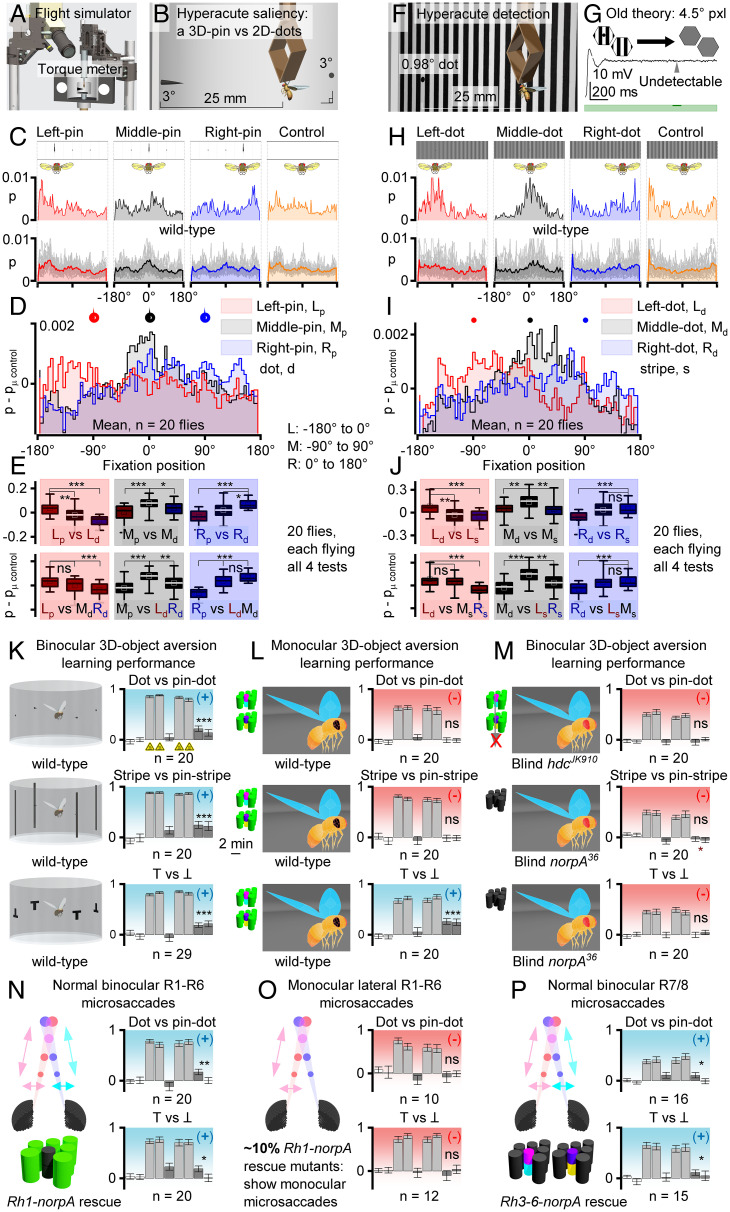
Hyperacute stereopsis requires two eyes with mirror-symmetric microsaccades. (*A* and *B*) In a flight simulator, a torque-meter–tethered flying *Drosophila* controls how a white cup rotates around it, showing three black dots (3.9° Ø), 90° apart, one with a black 4-mm center pin (1° Ø). Axially 25 mm away, the 3D pin at −90° (*Left*), 0° (*Middle*), or 90° (*Right*) dot is monocularly indistinguishable from the 2D dots. (*C*) Yet, flies fixate more on the pins than on the competing dots, implying 3D-pin salience. A single fly’s (*Above*) and population (*Below*) frontal fixation probability to the *Left*, *Middle*, and *Right* pin/dot positions, and during a blank control. (*D*) Fixation probabilities for the three pin positions, blank control subtracted to minimize experimental bias. (*E*) Positional salience (e.g., *Left* pin vs. *Left* dots, *Above*) and competition (e.g., *Left* pin vs. *Middle* and *Right* dots, *Below*) statistics indicate that *Drosophila* see hyperacute 3D pins among 2D dots (superresolution stereopsis). (*F*) Testing hyperacute 2D-object detection. (*G*) Old theory simulation: A fly with static 2D vision and 4.5° ommatidial pixelation cannot detect a black 0.98° dot hidden among 1.2° stripes, as its optically corrected contrast difference over a photoreceptor’s RF (5.4° half-width) is only ∼1.6% of that of the stripes alone, evoking response differences < voltage variation (noise) for such a contrast pulse (green, 100 ms). (*H*) Nevertheless, flies fixate on the hidden dot, irrespective of its position. A single fly’s (*Above*) and population (*Below*) frontal fixation probability to the *Left*, *Middle*, and *Right* dot positions, and during a stripe control. (*I*) Fixation probabilities for the three dot positions, stripe control subtracted to minimize bias. (*J*) Positional detection (e.g., *Left* dot vs. *Left* stripes, *Above*) and salience (e.g., *Left* dot vs. *Middle* and *Right* stripes, *Below*) statistics/trends indicate that *Drosophila* find hyperacute dots visually interesting. (*K*) *Drosophila* learns to avoid hyperacute 3D pins or 2D lines/dots (*Above* and *Middle*), associated with infrared (IR)-heat punishment (training, triangles), equally well to the classic T vs. ┴ conditioning (*Below*). (*L*) One-eye–painted *Drosophila* fails to learn hyperacute 3D- and 2D-object avoidance (*Above* and *Middle*), demonstrating that superresolution stereovision requires two eyes. Yet monocular *Drosophila* shows normal T vs. ┴ conditioning (*Below*), indicating that one eye is enough to learn large 2D patterns, consistent with retinal-position–invariant pattern recognition (51). (*M*) Blind *hdc^JK910^* (*Above*) and *norpA^36^* (*Middle* and *Below*), with no synaptic photoreceptor outputs but normal audition and olfaction, failed to learn the test stimuli, validating that the wild-type learning (*K* and *L*) was visual. (*N*) Rh1-rescue *norpA^36^* with functional R1–R6 photoreceptors and normal mirror-symmetric left and right eye microsaccades learned hyperacute 3D stimuli (*Above*) and large 2D patterns (*Below*), but less well than wild type. Thus, R7/R8s also contribute to stereopsis. (*O*) ∼10% of Rh1-rescue *norpA^36^* flies showed only left or right eye lateral microsaccade components, leading to asymmetric and asynchronous binocular sampling. These flies neither learned hyperacute 3D stimuli (*Above*) nor large 2D patterns (*Below*), meaning, mirror-symmetric microsaccadic sampling is necessary for hyperacute stereopsis. (*P*) *norpA^P24^* with rescued R7/R8 photoreceptors, showing normal microsaccades, learned to differentiate both coarse 2D and hyperacute 3D patterns. Thus, R7/R8s alone are sufficient for hyperacute stereopsis. Note, the exact microsaccadic movements during the experiments are unknown as it was too difficult to measure the DPP movement concomitantly.

To test whether *Drosophila* possesses superresolution stereovision, as our theory (Fig. 5 and *SI Appendix*, Fig. S59) predicts, we performed visual salience (*SI Appendix*, Figs. S69–S71 and Tables S7–S10) and learning experiments (*SI Appendix*, Figs. S72–S77 and Tables S11–S19) with hyperacute 3D and two-dimensional (2D) objects in a flight simulator system (Fig. 6). This apparatus was designed so that a tethered fly had no monocular cues to construct 3D representations of the objects neurally, without optically distorting its perception (*SI Appendix*, Fig. S68). In nature, flying insects typically keep an object of interest in frontal view, fixating it by small side-to-side head/body rotations (45, 46). Such movements, by modulating light input and thus mirror-symmetric microsaccades at the binocular eye regions, should accentuate 3D perception (Fig. 5). But conversely, given the photoreceptor RF dynamics and binocular separation, 3D perception must diminish with increasing distance, as sampling uncertainties increase, predicting ∼3- to 70-mm hyperacute stereo range (Fig. 5*E* and *SI Appendix*, Fig. S59). Therefore, we presented stimuli 25 mm from the eyes, well within this range.

In salience experiments, a tethered flying fly explored a white panoramic scene, which had a small (4-mm-long) black hyperacute (i.e., <4.5° interommatidial pixelation) (12) 3D pin, protruding from a small black dot (3.9° Ø), and two similar-sized black 2D dots, each 90° apart (Fig. 6 *A*–*C*). The pin position was varied for three trials, and the fourth (control) was a blank scene, presented in random order. For each trial, we measured a fly’s fixation behavior: How much time it kept each part of the scene at the fontal view, given as probability. The conventional compound eye acuity theory (8, 9) states that because all these three objects had the same contrast and were smaller than the eyes’ interommatidial pixelation, their differences would be invisible, giving them equal salience, and *Drosophila* should fixate all three of them with equal probability. Whereas, our mirror-symmetric microsaccadic sampling theory (Fig. 5 and *SI Appendix*, Fig. S59) predicts that for a fly with hyperacute 3D vision, the 3D pin would appear different from the 2D dots, with its saliency increasing fixations. In supporting our theory, the results showed that *Drosophila* prefers to fixate hyperacute 3D pins, irrespective of their positioning (Fig. 6 *C*–*E*). Equally, in separate experiments, the flies readily fixated on hyperacute 2D dots (0.98°) hidden in a 1.0° hyperacute stripe scene (Fig. 6 *F*–*J*), which by the conventional theory would be impossible (Fig. 6*G*). Moreover, the flies’ optomotor responses to hyperacute stimulation (*SI Appendix*, Figs. S63–S65) followed the predictions of our theory (*SI Appendix*, Figs. S66 and S67).

In learning experiments (Fig. 6*K*), *Drosophila* saw both hyperacute 2D objects (black bars, above or dots, middle) and hyperacute 3D objects (black pins inside bars or dots) and were trained by associative heat punishment (*SI Appendix*, Fig. S73) to avoid one or the other stimulus. Again, in support of our theory, the flies readily learned to avoid the punishment-associated stimulus, validating that they saw hyperacute 3D objects differently from their 2D counterparts (of the same area/contrast). This learning was robust, matching the classic large-pattern T vs. ┴ performance (47) (below). But importantly, it was abolished when either the left or the right eye was painted black (Fig. 6 *L*, *Above* and *Middle*), indicating that hyperacute 3D vision requires inputs from both eyes. In contrast, the large-pattern T vs. ┴ learning still occurred with one eye only (*Below*), consistent with the reported retinal position invariance in visual pattern recognition (47). Whereas, blind *hdc^JK910^* (Fig. 6 *M*, *Above*), *norpA^36^* (*Middle* and *Below*) mutants, having no synaptic photoreceptor outputs but functioning auditory and olfactory senses, failed to learn the test stimuli. These results corroborate wild-type *Drosophila* seeing the nearby world and learning its objects in hyperacute stereo.

Finally, we tested whether learning hyperacute 3D stimuli requires either R1–R6 or R7/R8 photoreceptors or both with intact microsaccadic sampling. Here, we exploited our serendipitous finding that rescuing R1–R6 or R7/R8 photoreceptors in blind *norpA^P24^* mutants makes their microsaccades’ lateral component more fragile to mechanical stress or developmentally imperfect, with not every tethered fly showing them (*SI Appendix*, Fig. S74). Therefore, after the learning experiments, we recorded each fly’s light-induced deep pseudopupil movement (Fig. 3) and ERG, quantifying their microsaccades and phototransduction function, respectively. We found that while most *norpA^P24^* Rh1-rescue flies (R1–R6s are sampling, R7/R8s not) showed normal binocular microsaccades (Fig. 6*N*), ∼10% showed microsaccades only monocularly (Fig. 6*O*). Importantly, however, each fly eye (both left and right) showed a characteristic ERG, indicating that its phototransduction, and thus axial microsaccade movement from PIP_2_ cleavage (10, 11), was unspoiled. The flies with normal lateral microsaccades (Fig. 6*N*) learned the difference between hyperacute pins and dots (*Above*) and large T vs. ┴ patterns (*Below*), but less well than wild-type flies (Fig. 6*K*), establishing that R1–R6 input is sufficient for hyperacute stereovision but that R7/R8s must also contribute. Conversely, the flies that showed monocular lateral microsaccades (Fig. 6*O*) neither learned hyperacute 3D objects (*Above*) nor large 2D patterns (*Below*), indicating that misaligned binocular sampling corrupts 3D perception and learning. Whereas R7/R8 rescued *norpA^P24^* (Fig. 6*P*) and *ninaE^8^* mutants confirmed that the inner photoreceptors also contribute to hyperacute stereopsis.

These findings concur with our simulation results, which predicted that asynchronous binocular sampling should break stereopsis (*SI Appendix*, Fig. S60). Collectively, these results demonstrate that binocular mirror-symmetric microsaccadic sampling is necessary for superresolution stereovision and that both R1–R6 and R7/R8 photoreceptor classes contribute to it.

## Discussion

We showed how the *Drosophila* compound eyes’ binocular mirror-symmetric photoreceptor microsaccades (Figs. 1–3) generate phasic disparity signals in much finer resolution than ommatidial pixelation, suggested by their interommatidial angle (Figs. 4 and 5). The fly brain could use these signals to triangulate object distance to a neural distance signal in time (Fig. 5), enabling stereopsis (Fig. 6). We also revealed how a flying fly’s optic flow field affects the microsaccadic sampling across the eyes to enhance visual information capture from the world in motion (Figs. 3 and 4). Visual behavior matched the modeling predictions (Figs. 5 and 6), demonstrating that the neural image generated by mirror-symmetric microsaccadic sampling must result in a higher-quality perceptual representation of the stimulus as compared to the neural image generated by immobile photoreceptors (8, 9) or asymmetric or asynchronous binocular sampling (*SI Appendix*, Fig. S60). By integrating in vivo assays from subcellular to whole animal 3D perception with multiscale modeling from adaptive optics to depth computations (*SI Appendix*, Figs. S49–S61), these results establish a morphodynamic light information sampling and processing theory for compound eyes to better understand insect vision and behaviors (11, 48). To further demonstrate its explanatory power, we also verified its predictions of *Drosophila* seeing nearby objects in higher resolution (*SI Appendix*, Fig. S66) and “optomotor behavior reversal” (49) resulting, not from spatial aliasing as widely believed, but rather from the mirror-symmetric microsaccadic sampling of the left and right eyes (*SI Appendix*, Fig. S67).

It has long been thought that because the eye and head movements are dominated by axial rotation, they should provide little distance information as objects, near and far, would move across the retina with the same speed (50). In contrast, our study highlights how the visual systems can use microsaccades, and eye/head rotations, to both contrast enhance (*SI Appendix*, Fig. S26 and Movie S7) and extract depth information (Movie S10). Rapid mirror-symmetric inward-rotating photomechanical photoreceptor microsaccades in the left and right eyes, sampling the frontal binocular field of view, cause phase-difference signals, which inform the *Drosophila* brain in time how far an object is from its eyes. But when the world is still, a fly can further contract its intraocular muscles (19), rotate or move its head from side to side, as insects with compound eyes commonly do during fixation, to generate both binocular and motion parallax (51) signals to resolve object depth.

With mirror-symmetric microsaccadic sampling, flies and possibly other insects with binocular compound eyes can have an intrinsic sense of size. For two objects with equal angular size and velocity as projected on the eyes, the closer one, and thus physically smaller (a mate), generates a brief and precise binocular disparity in time, while the other object, further away and thus bigger (a predator), generates longer-lasting but more blurred disparity.

This encoding strategy applies to machine vision. Superresolution depth information about a nearby object (moving or still) can be extracted in time, for example, by piezo resonating synchronously and mirror symmetrically two horizontally separated sampling matrixes (left and right) with overlapping views and then correlating their phasic differences for each corresponding pixel. This procedure equates to a two-matrix extension of the VODKA (Vibrating Optical Device for the Kontrol of Autonomous robots) sensor principle (52). In more sophisticated optic flow-optimized 3D systems, binocular photomechanical pixel sensors could move along their specific concentric rotation axes as in the *Drosophila* eyes.

We note that recent work has shown that human cones (53) and vertebrate rod photoreceptors (54) contract photomechanically, comparable to *Drosophila* photoreceptor microsaccades (10, 11). It will be interesting to see whether these microsaccades increase visual acuity and participate in stereovision and whether high-intensity X-rays also activate them (13–15).

## Materials and Methods

The multiscale experimental and theoretical approaches used in this study are explained in detail in *SI Appendix*, organized in sections I through VIII. Fruit flies (*Drosophila melanogaster*) were raised at 18 °C in a 12-h/12-h dark/light cycle. As listed in *SI Appendix*, Section VIII, wild-type, various mutant and transgenic stocks of female and male flies were used. The flies’ photomechanical photoreceptor microsaccades were studied in vivo using high-speed X-ray and infrared microscopy, following the preparation and dissection protocols as explained in *SI Appendix*, Sections I–III. High-speed, high-resolution Ca^2+^-imaging of lamina L2-neurons’ responses to hyperacute visual stimulation was done in vivo using 2-photon imaging as described in *SI Appendix*, Section IV. The mathematics behind multiscale modeling of the adaptive optics and photoreceptor signaling are detailed in *SI Appendix*, Section V and the anatomical rational in *SI Appendix*, Section VI. The *Drosophila* flight simulator experiments are explained in *SI Appendix*, Section VII.

## Supplementary Material

Supplementary File

Supplementary File

Supplementary File

Supplementary File

Supplementary File

Supplementary File

Supplementary File

Supplementary File

Supplementary File

Supplementary File

Supplementary File

## Data Availability

X-ray imaging data have been deposited in GitHub (https://github.com/JuusolaLab/Hyperacute_Stereopsis_paper). All other study data are included in the article and/or supporting information.
